# Comparative Analysis of Clinical-Scale IFN-γ-Positive T-Cell Enrichment Using Partially and Fully Integrated Platforms

**DOI:** 10.3389/fimmu.2016.00393

**Published:** 2016-09-30

**Authors:** Christoph Priesner, Ruth Esser, Sabine Tischer, Michael Marburger, Krasimira Aleksandrova, Britta Maecker-Kolhoff, Hans-Gert Heuft, Lilia Goudeva, Rainer Blasczyk, Lubomir Arseniev, Ulrike Köhl, Britta Eiz-Vesper, Stephan Klöß

**Affiliations:** ^1^Institute of Cellular Therapeutics, Hannover Medical School, Niedersachsen, Germany; ^2^Integrated Research and Treatment Center Transplantation (IFB-Tx), Hannover Medical School, Niedersachsen, Germany; ^3^Institute for Transfusion Medicine, Hannover Medical School, Niedersachsen, Germany; ^4^Department of Pediatric Hematology and Oncology, Hannover Medical School, Niedersachsen, Germany

**Keywords:** closed GMP-compliant systems, immunoaffinity cell selection, virus-specific T-cells, single platform and multicolor flow cytometry, CliniMACS Cytokine-Capture-System

## Abstract

**Background and aims:**

The infusion of enriched CMV-specific donor T-cells appears to be a suitable alternative for the treatment of drug-resistant CMV reactivation or *de novo* infection after both solid organ and hematopoietic stem cell transplantation. Antiviral lymphocytes can be selected from apheresis products using the CliniMACS Cytokine-Capture-System^®^ either with the well-established CliniMACS^®^ Plus (Plus) device or with its more versatile successor CliniMACS Prodigy^®^ (Prodigy).

**Methods:**

Manufacturing of CMV-specific T-cells was carried out with the Prodigy and Plus in parallel starting with 0.8–1 × 10^9^ leukocytes collected by lymphapheresis (*n* = 3) and using the MACS GMP PepTivator^®^ HCMVpp65 for antigenic restimulation. Target and non-target cells were quantified by a newly developed single-platform assessment and gating strategy using positive (CD3/CD4/CD8/CD45/IFN-γ), negative (CD14/CD19/CD56), and dead cell (7-AAD) discriminators.

**Results:**

Both devices produced largely similar results for target cell viabilities: 37.2–52.2% (Prodigy) vs. 51.1–62.1% (Plus) CD45^+^/7-AAD^−^ cells. Absolute numbers of isolated target cells were 0.1–3.8 × 10^6^ viable IFN-γ^+^ CD3^+^ T-cells. The corresponding proportions of IFN-γ^+^ CD3^+^ T-cells ranged between 19.2 and 95.1% among total CD3^+^ T-cells and represented recoveries of 41.9–87.6%. Within two parallel processes, predominantly IFN-γ^+^ CD3^+^CD8^+^ cytotoxic T-cells were enriched compared to one process that yielded a higher amount of IFN-γ^+^ CD3^+^CD4^+^ helper T lymphocytes. T-cell purity was higher for the Prodigies products that displayed a lower content of contaminating IFN-γ^−^ T-cells (3.6–20.8%) compared to the Plus products (19.9–80.0%).

**Conclusion:**

The manufacturing process on the Prodigy saved both process and hands-on time due to its higher process integration and ability for unattended operation. Although the usage of both instruments yielded comparable results, the lower content of residual IFN-γ^−^ T-cells in the target fractions produced with the Prodigy may allow for a higher dosage of CMV-specific donor T-cells without increasing the risk for graft-versus-host disease.

## Introduction

The adoptive transfer of allogeneic CMV-specific T-cells gains clinical importance in the selective elimination of the virus as an intervention in CMV infections refractory to conventional antiviral treatment in hematopoietic stem cell transplantation (HSCT) and solid organ transplantation (SOT) (1). The absence and/or delayed onset of *in vivo* proliferation of CMV-specific T-cells during immune reconstitution in SCT can lead to critical conditions of patients facing CMV reactivation or *de novo* infection. Correspondingly, the continual immunosurveillance by active CMV-specific T-cells as a basic requirement in these patients plays an essential role for a relevant clearance of early and late CMV infections and reactivations before and after days 90–100 post-HSCT (2–4).

While the overall response time between therapeutic decision and start of therapy impacts therapeutic success, its duration depends on both the availability of an eligible and consenting donor and the rapid generation of the cellular therapeutic. Different strategies for the preparation of allogeneic antigen-specific T-cells for adoptive transfer have been investigated and developed to clinical scale so far (5–10). One promising rapid technique involves the short-term *ex vivo* restimulation of virus-specific T-cells from lymphapheresis products collected from seropositive donors, followed by the isolation of antiviral T-cells based on the secretion of interferon-γ (IFN-γ). Following specific activation with defined viral antigens such as pp65, T-cells release IFN-γ that is finally targeted for immunomagnetic enrichment of the activated T-cell subsets (5, 6, 8, 11). The use of pooled synthetic overlapping peptides covering the primary structure of the viral antigen results in a wide diversity of antiviral CD4^+^ and CD8^+^ T-cell subsets, thus overcoming the HLA restriction that is characteristic of the second rapid method, the reversible major histocompatibility complex (MHC) class I multimer technology (5, 12).

The feasibility and compliance to the requirements of good manufacturing practices (GMP) of the *ex vivo* restimulation, immunomagnetic labeling, and enrichment of antigen-specific T-cells outlined above in clinical scale have been demonstrated using MACS^®^ GMP PepTivators (e.g., HCMV pp65) and the CliniMACS Cytokine-Capture-System^®^ (CCS^®^) both, developed and commercialized by Miltenyi Biotec GmbH (9, 13, 14).

The reagent system is intended to be used with a platform technology of microprocessor-controlled instruments that provide for semi-automated thus standardized cell processing in disposable closed systems, the well-established CliniMACS^®^ Plus device (Plus) and its refined successor, the CliniMACS^®^ Prodigy^®^ device (Prodigy). Since the process management and control of the Plus device is limited to the liquid handling of intermediates and reagents during the immunomagnetic enrichment, process steps sensitive to time, temperature, and temperature profile have to be operated hands-on offline. With its added temperature-controlled centrifugation and cell-culture capabilities, the Prodigy allows for the integration of the whole manufacturing process in one device promising increased precision while reducing hands-on time. In the present study, we compare our results of and experiences with the application of the CCS^®^ protocol for the generation of clinical-grade CMV-specific T-cells with the Prodigy to those we gathered with the Plus as previously published (14), focusing on inter-instrument precision by applying established quality control (QC) protocols.

## Materials and Methods

In order to compare the devices, lymphapheresis product was split and 0.8–1 × 10^9^ WBC each were processed on both instruments in parallel. The procedures for donor recruitment, lymphapheresis, and selection of the IFN-γ-positive CMV-specific T-cells with the Plus device are thoroughly described elsewhere (14). Thus, only brief summaries thereof are shown below.

### Recruitment of CMV-Reactive T-Cell Donors and Cell Collection

Medically eligible and specifically suitable donors were recruited from alloCELL, the allogeneic T-cell donor registry of Hannover Medical School’s (MHH) Institute for Transfusion Medicine as previously described (14). Briefly, upon written informed consent, >3 × 10^9^ leukocytes were collected by lymphapheresis (LA) from three donors, each seropositive for anti-CMV IgG, seronegative for anti-CMV IgM, and exhibiting frequencies of >0.03% IFN-γ^+^/CD3^+^ CMV-specific memory T-cells in the peripheral blood (PB) and their adequate answer to restimulation as established by IFN-γ Enzyme-linked ImmunoSpot Assay (ELISpot) and MACS^®^ IFN-γ Cytokine Secretion Assay (CSA).

### Clinical Grade of CMV-Specific T-Cell Selection

IFN-γ-secreting CD3^+^ T-cells specific against peptides covering the HCMV pp65 antigen were restimulated and enriched in compliance with EU GMP using the Plus instrument within the legally required manufacturing validation (14), whereas the Prodigy runs were carried out within pre-GMP process development. In both the settings, the CliniMACS CCS^®^ system including reagents and consumables was used following the manufacturer’s written instructions (14).

**Figure 1:**
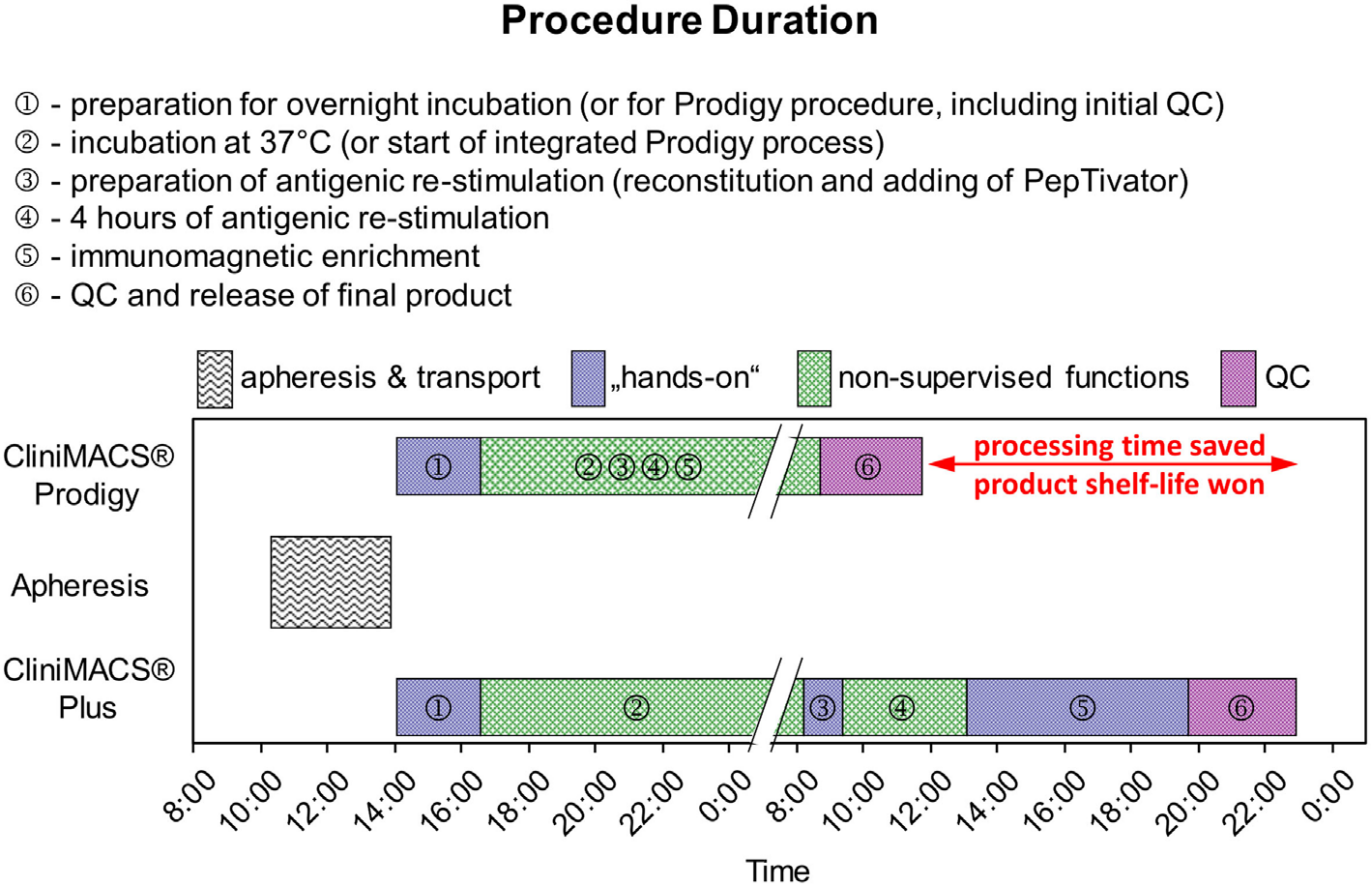
**Schematic comparison of manufacturing processes for CMV-specific T-cells using CliniMACS^®^ Plus and CliniMACS Prodigy^®^**. Both processes are largely comparable for washing, incubation, and centrifugation steps during CCS procedures and the immunomagnetic enrichment of IFN-γ-secreting leukocytes. However, the processes *via* Plus procedure required additional labor steps resulted in a time delay to the next morning. Thus, the Prodigy process performance revealed a limited hands-on time period after the process initiation.

The collected LAs were split and processed in parallel on the CliniMACS^®^ Plus device and the Prodigy^®^ instrument. For the Plus, 2 × 10^9^ nucleated blood cells were washed for platelet reduction (15 min 200 *g*, ambient temperature, TexMACS^®^ GMP medium, Miltenyi Biotec), suspended in 200 ml of the same medium and stored overnight in 500ml MACS^®^ GMP Cell Differentiation Bags (Miltenyi Biotec) at 37°C and 5% CO_2_. Of these, 1 × 10^9^ viable nucleated cells were used for the restimulation and immunoaffinity enrichment procedure as previously described (14). In contrast, the preparation with the Prodigy was initiated immediately with 1 × 10^9^ viable nucleated cells from the native lymphapheresis. *Ex vivo* restimulation (4 h, 37°C, 5% CO_2_) with the GMP-grade CMVpp65 peptide pool (MACS^®^ GMP PepTivator^®^ HCMV pp65, 1 μg/ml per peptide, Miltenyi Biotec), labeling of WBCs with the CliniMACS CCS^®^ Catchmatrix Reagent (37°C, 5% CO_2_), cooling and cooled centrifugation steps (2–6°C), and labeling were carried out manually with the Plus and autonomously within the Prodigy instrument. Finally, the enrichment of IFN-γ-secreting cells was performed *via* immunomagnetic separation (Plus and Prodigy) by antibody-conjugated super-paramagnetic particles (CliniMACS IFN-γ Enrichment Reagent, Miltenyi Biotec). All washing, incubation, and centrifugation steps during CCS^®^ processes on both separation devices utilized CliniMACS PBS/EDTA buffer. Figure 1 summarizes the manufacturing steps of the comparative procedures.

### Quality Control: Cell Enumeration and Phenotypic Analysis

The QC applied to the validation runs with the Plus system was performed according to the correspondingly validated standard operating procedures as shown by Tischer et al. (14). Briefly, the estimation of viability (trypan blue exclusion) and number of cells constituting the comparatively cell-poor target fraction [positive fraction (PF)] was carried out microscopically. The other fractions, e.g., LA, preselection (PreS) were defined as the cell fraction after restimulation and labeling immediately before immunomagnetic separation, and the negative fraction (NF) were analyzed for viability by flow cytometry [7-aminoactinomycin D (7-AAD)], whereas cell enumeration was performed with an automated Hemocytometer (Coulter ACTdiff, Beckman Coulter). For phenotyping, six-color flow cytometry (BD FACSCanto™ II, BD Biosciences) was used in this dual-platform setting.

**Figure 2:**
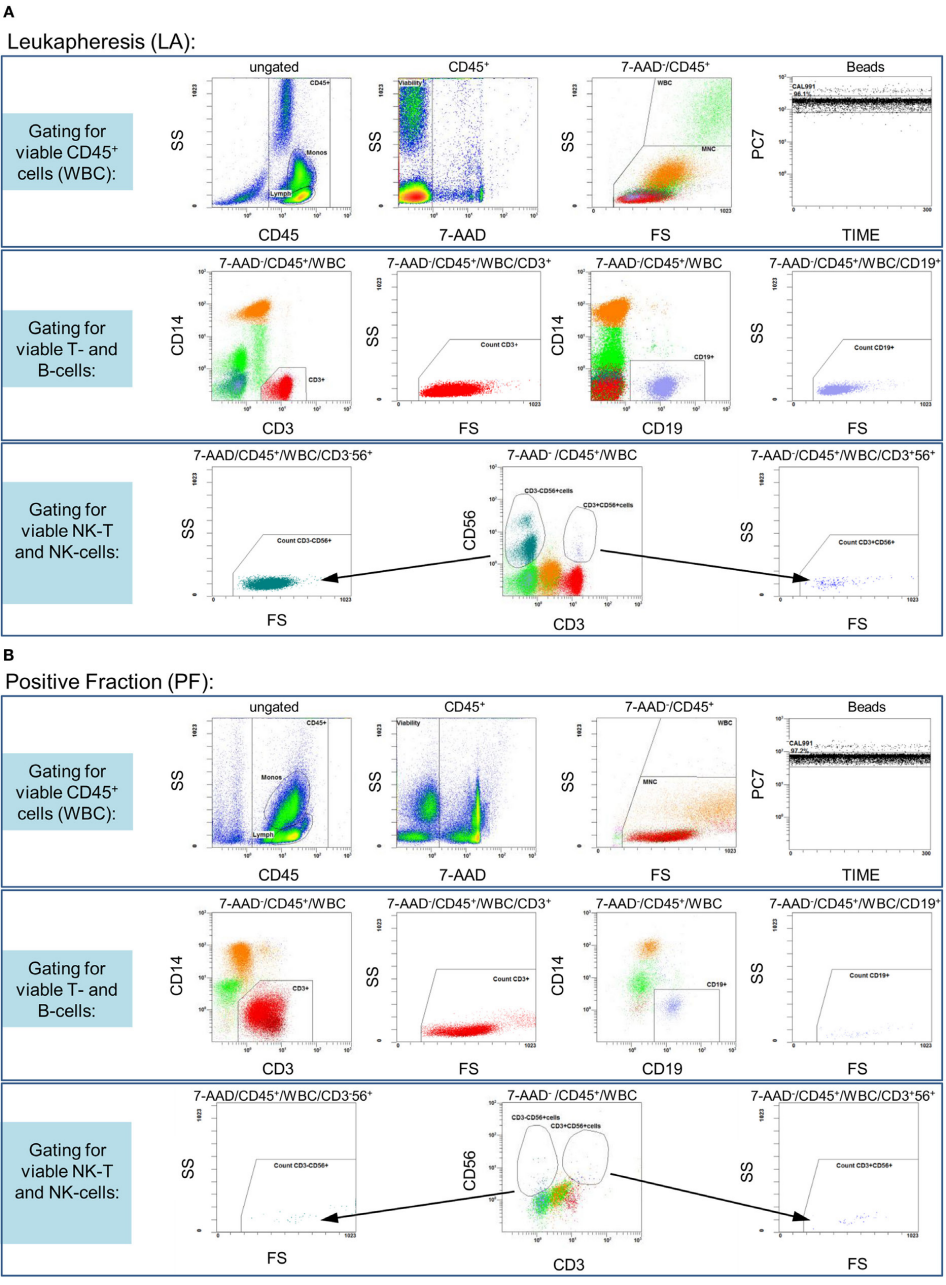

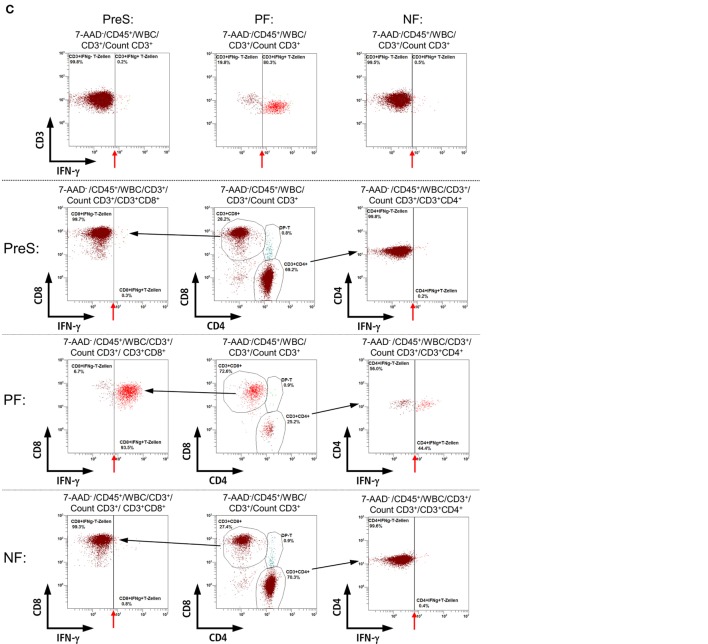
**Gating strategy: flow cytometric quality control for the quantification of IFN-γ^+^ and IFN-γ^−^ (CD3^+^/CD4^+^/CD8^+^) T-cells**. Samples from different process fractions (LA, PreS, PF, and NF) were stained with monoclonal antibodies to detect IFN-γ^+^ (CD3^+^/CD4^+^/CD8^+^) T-cells and analyzed by a no-wash, single-platform procedure based on a nine-color flow cytometric protocol. Therefore, CD45^−^, 7-AAD^+^, and non-specifically stained debris assessed by low forward and side scatter (FS/SS) signals were excluded from viable CD45^+^ cells (WBC). One histogram illustrates the events of the region “beads” along the time course to calibrate the events for cells/μl and detect even sample flow (**A,B**, LA, and PF, upper rows). For the detection of T- and B-cells, the pre-analyzed viable CD45^+^ cells were used to identify both lymphocyte subsets based on CD3 or CD19 surface expression. Therefore, FS/SS dot plots are linked with the region “MNC” (upper row) to exclude debris and outline the correct lymphocyte regions (**A,B**, LA, and PF, middle rows). Dump channels allowed to separate (CD3 vs. CD56) NK-T (CD3^+^CD56^+^) and NK (CD3^−^CD56^+^) cells (black arrows) from the viable CD45^+^ cells (especially viable CD56^−^CD3^+^ T-cells) to identify IFN-γ^+^ (CD3^+^/CD4^+^/CD8^+^) T-cells (**A,B**, LA, and PF, lower rows). Exemplarily shown by the analyses of PreS, PF, and NF of one manufacturing process is the bordering between IFN-γ^−^ and IFN-γ^+^ (CD3^+^/CD4^+^/CD8^+^) T-cells (indicated with red arrows, **C**). To distinguish between CD4^+^ and CD8^+^ T-cell subsets, the viable CD56^−^CD3^+^ T-cells (**A,B**) were separated by dump channel in CD4^+^ and CD8^+^ T-cells (black arrows, **C**) followed by separations of IFN-γ^−^ and IFN-γ^+^ (CD4^+^/CD8^+^) T-cell subsets.

In accordance with the requirements of the European Pharmacopoeia’s monography for the “numeration of CD34/CD45^+^ cells in hematopoietic products” (EP 2.7.23) a new no-wash, single-platform 9-color flow cytometric assay was developed to analyze the different cell fractions on a 10-color flow cytometer (Navios, Beckman Coulter) resulting from process establishment runs on the Prodigy. *In vitro* diagnostic (IVD)- and/or analyte-specific reagent (ASR)-labeled, fluorescence-conjugated monoclonal antibodies (mABs), especially anti-CD3 [phycoerythrin-cyanin-7 (PC-7)], anti-CD56 (phycoerythrin-Texas Red, ECD), anti-CD8 [allophycocyanin (APC)], anti-CD19 (APC A700), anti-CD4 (APC A750), anti-CD14 (Pacific Blue), and anti-CD45 (Krome Orange), were utilized for the phenotyping of different leukocyte subpopulations by flow cytometric analysis. All mABs were purchased from Beckman Coulter with the exception of IFN-γ [phycoerythrin (PE); Miltenyi Biotec] for the detection of the IFN-γ-labeled target cells. Additionally, dead cells were identified by 7-AAD staining in the same panel, whereas cell debris was discriminated by the cells’ forward scatter (FS) and side scatter (SS) properties as well as by adjusting the cell discriminator (similar to the threshold function of other manufacturers’ software) integrated in the Beckman Coulter software (Navios™ Cytometry List Mode Data Acquisition and Analysis software). After the labeling of samples with multiple mABs (15 min, ambient temperature) and subsequent red blood cell lysis (15 min, ambient temperature, IOTest 3, Beckman Coulter) with 5,000–10,000 cells per microliter, total event numbers of at least 100,000 (cell-rich LA, PreS, and NF) or 10,000 (cell-poor PF) were acquired for the 7-AAD^−^ and CD45^+^ leukocyte regions (Figure 2). Finally, the IFN-γ^+^ (CD4^+^/CD8^+^) T-cells and contaminating IFN-γ^−^ leukocyte subsets were analyzed.

### Statistical Analysis

For graphical and statistical evaluation, the GraphPad Prism software v6.02 (GraphPad, San Diego, CA, USA) was used. Data sets of all runs were analyzed individually for recovery, restimulation frequencies, residual/contaminating leukocytes, and viability of target and non-target cells in the different fractions and compared between corresponding runs on both instruments. Unless otherwise declared, results of the statistical evaluations are indicated as median with range within the individual text modules.

## Results

### Selection of CMV-Specific T-Cells Using the New Fully Automated Prodigy

The feasibility of clinical-scale manufacturing of CMV-specific T-cells using the Prodigy instrument could be shown successfully. A novel single-platform nine-color flow cytometry protocol was established in order to quantify CMV-specific IFN-γ^+^ T-cells and various leukocyte subpopulations in the different fractions (LA, PreS, PF, and NF) during the process as exemplarily shown in Figure 2.

The quantification of PFs resulted a median yield of 1.5 × 10^6^ (range: 0.21–9.6) of total viable CD45^+^ cells and 0.9 × 10^6^ (range: 0.14–4.8) of total viable CD3^+^ T-cells over all process runs (Table 1). The range of viable IFN-γ^+^CD4^+^ T-cells and IFN-γ^+^CD8^+^ T-cells was measured between 0.01–0.7 × 10^6^ and 0.1–3.1 × 10^6^ cells, respectively. Additional parameters characterizing PF and LA of the three Prodigy processes are compiled for individual comparison in Table 1.

**Table 1:** **Comparison of flow cytometric cell counts and subset ratios for three clinical-scale CliniMACS Prodigy^®^ procedures using the CliniMACS CCS^®^ protocol for the manufacture of CMV-specific T-cells**.

LA → PF	CliniMACS Prodigy^®^								
	Run 1	Run 2	Run 3
**LA**			
Viable CD45^+^ cells [×10^9^]	1.0	0.8	1.0
Viable CD3^+^ [×10^6^]	486.2	300.1	167.3
CD4/CD8 ratio (viable CD3^+^)	1.2	2.0	1.02
**PF**			
Viable CD45^+^ cells [×10^6^]	9.6	0.21	1.5
Viable CD3^+^ [×10^6^]	4.8	0.14	0.9
CD4/CD8 ratio (viable CD3^+^)	0.32	0.26	6.64
Viable IFN-γ^+^CD3^+^ [×10^6^]	**3.8**	**0.12**	**0.9**
Viable IFN-γ^−^CD3^+^ [×10^6^]	1.1	0.01	0.04
CD4/CD8 ratio (viable IFN-γ^+^CD3^+^)	0.2	0.1	6.2
Viable IFN-γ^+^ CD3^+^CD4^+^ [×10^6^]	**0.6**	**0.01**	**0.7**
Viable IFN-γ^−^CD3^+^CD4^+^ [×10^6^]	0.4	0.01	0.03
Viable IFN-γ^+^ CD3^+^CD8^+^ [×10^6^]	**3.1**	**0.1**	**0.1**
Viable IFN-γ^−^CD3^+^CD8^+^ [×10^6^]	0.3	0.01	0.01

*Absolute numbers [×10^6^] of viable CD45^+^ cells, IFN-γ^+^ and IFN-γ^−^ T-cells (CD3^+^/CD4^+^/CD8^+^), and T-cell ratios (CD4^+^/CD8^+^) of lymphapheresis (LA) and positive fractions (PFs) derived from three healthy CMV seropositive donors displayed individually for each manufacturing run*.

*Absolute yields of target cells were 0.1–3.8 × 10^6^ viable IFN-γ+CD3+ T-cells subdivided into IFN-γ+CD4+ T-cells (0.01–0.7 × 10^6^) and IFN-γ+CD8+ T-cells (0.1–3.1 × 10^6^) (bold numbers)*.

**Figure 3:**
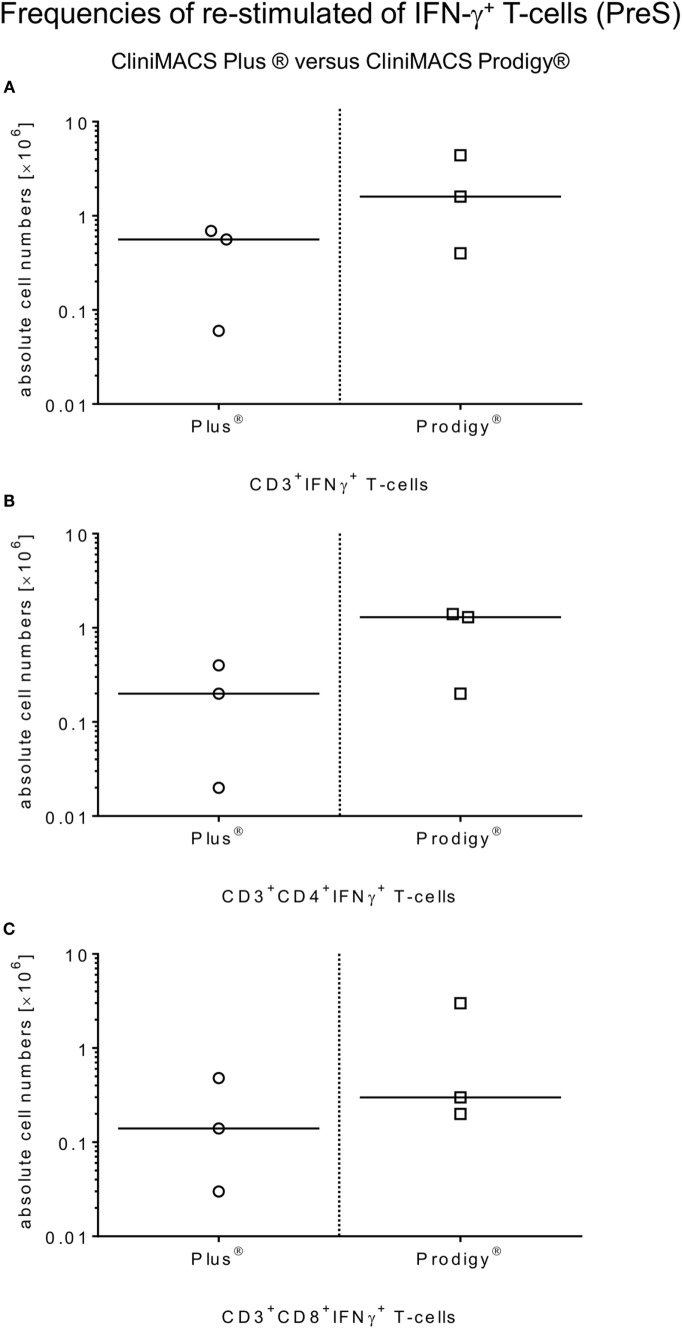
**Comparison of *ex vivo* restimulation of CMV-specific T-cells by antigenic restimulation by platform**. Restimulation frequencies of CMV-specific T-cells were compared between all manufacturing processes of Plus and Prodigy. Samples were collected from the PreS fractions after incubation (4 h, 37°C, 5% CO_2_) with the HCMVpp65 peptide pool (MACS GMP PepTivator HCMV pp65, 1 μg/ml per peptide). Flow cytometric analyses were performed to identify total IFN-γ^+^-releasing CD3^+^ T-cells **(A)** and (CD4^+^/CD8^+^) T-cell subsets **(B,C)** in those PreS fractions to compare both restimulation procedures.

The restimulation of donor-derived CMV-specific T-cells by antigen incubation was quantified by calculating the ratio of absolute numbers [×10^6^] of IFN-γ^+^CD3^+^ T-cells and (CD4^+^ and CD8^+^) T-cell subsets of PreS fractions and the corresponding T-cell fractions of the non-stimulated LA (PreS:LA = restimulation factor) as indicated in Table 2 for IFN-γ^+^ (CD3^+^/CD4^+^/CD8^+^) T-cells. The median total cell numbers of IFN-γ^+^CD3^+^ T-cells and (CD4^+^ or CD8^+^) T-cell subsets were as follows: IFN-γ^+^CD3^+^ T-cells: 1.6 × 10^6^, range: 0.4–4.4; IFN-γ^+^CD3^+^CD4^+^ T-cells: 1.3 × 10^6^, range: 0.2–1.4; and IFN-γ^+^CD3^+^CD8^+^ T-cells: 0.3 × 10^6^, range: 0.2–3.0, respectively (Figure 3).

**Table 2:** **Results of antigenic restimulation *ex vivo* of CMV-specific T-cells by Plus and Prodigy procedure**.

LA → PreS	CliniMACS^®^ Plus			CliniMACS Prodigy^®^		
	Run 1	Run 2	Run 3	Run 1	Run 2	Run 3
*x*-fold CD3^+^IFN-γ^+^	6.3	0.2	4.3	44.2	1.4	16.0
*x*-fold CD4^+^IFN-γ^+^	2.5	0.1	4.0	35.0	1.0	13.1
*x*-fold CD8^+^IFN-γ^+^	13.7	0.3	28.6	12.3	5.3	30.0

*The restimulation factors for CMV-specific T-cells were determined for each run by the ratio of flow cytometric total viable IFN-γ^+^ (CD3^+^/CD4^+^/CD8^+^) T-cells of non-stimulated lymphapheresis (LA) and PreS fractions (after *ex vivo* incubation, restimulation, and labeling prior to immunomagnetic enrichment). Restimulation carried out using a GMP-grade HCMVpp65 peptide pool (MACS GMP PepTivator HCMV pp65, 1 μg/ml per peptide)*.

### CMV-Antigen-Specific T-Cell Separation Using Plus Compared to Prodigy

Both Plus and Prodigy procedures were assessed with respect to yield and efficiency of the enrichment of CMV-responsive T-cells. Cell recovery, viability, and numbers of IFN-γ^+^ and IFN-γ^−^ T-cells, and T-cell subsets were analyzed in all process fractions.

**Figure 4:**
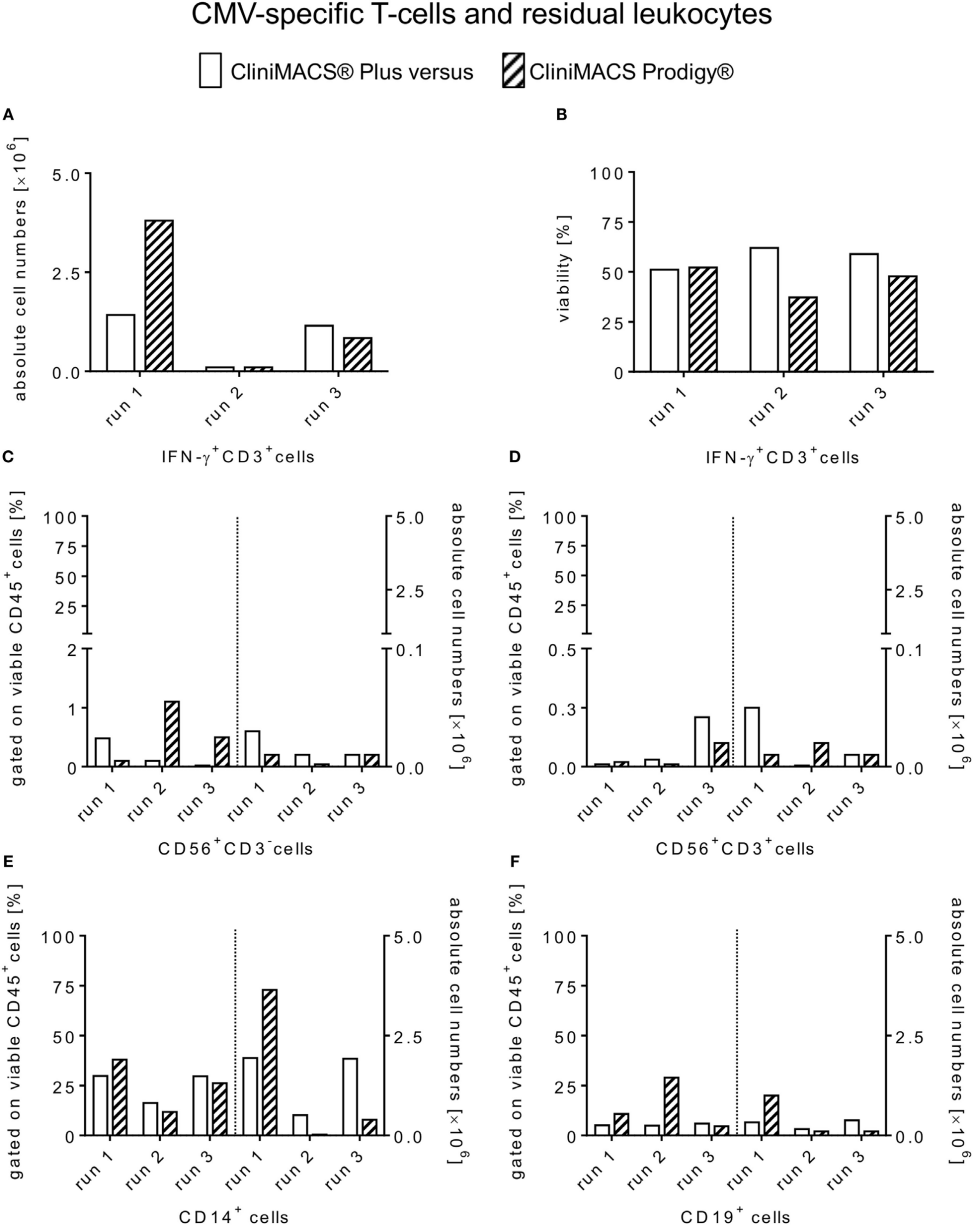
**Comparison of absolute cell counts and viability of enriched CMV-specific T-cells and contaminating residual non-target leukocytes**. Flow cytometry data of positive fractions from all three healthy CMV-positive donors are shown for each manufacturing run of CMV-specific T-cells selection using the CliniMACS^®^ Plus and Prodigy^®^ procedures. Individual values are presented for both platforms including **(A)** the total numbers [×10^6^] of viable IFN-γ^+^ (CD3^+^) T-cells, **(B)** cell viability of PF (%) and residual non-target subsets in relation to CD45^+^/7-AAD^−^ cells in the PF [(%) **(C–F)**].

Starting with a median of 1.6 × 10^6^ viable IFN-γ^+^CD3^+^ T-cells in the PreS fraction (Figure 3A), the immunomagnetic separation by Prodigy led to 0.9 × 10^6^ (range: 0.1–3.8) IFN-γ^+^CD3^+^ T-cell (Figure 4A) corresponding to a median recovery of 54.4% (range: 41.3–87.6%) compared to the median recovery of 77.7% (range: 41.9–83.9%) over all Plus processes (Table 3). The purity calculated as the percentage of IFN-γ^+^CD3^+^ T-cells of total CD3^+^ T-cells in the final PF products showed a median of 84.8% (range: 78.2–95.1%, calculated from Table 1) for the Prodigy processes compared to a median of 63.1% (range: 19.2–81.2%) in the Plus runs (14), respectively. Moreover, the medians of viability for recovered CD45^+^ cells were comparable for both procedures: median of Prodigy: 47.8% (range: 37.2–52.2%) and median of Plus processes 58.9% (range: 51.1–62.1%) as depicted in Figure 4B.

**Table 3:** **Comparative findings of both Plus and Prodigy process runs for the separations of CMV-specific T-cells**.

PreS → PF	CliniMACS^®^ Plus			CliniMACS Prodigy^®^		
	Run 1	Run 2	Run 3	Run 1	Run 2	Run 3
CD3^+^IFN-γ^+^ recovery (%)	83.9	41.9	77.7	87.6	41.3	54.4
CD4^+^IFN-γ^+^ recovery (%)	69.9	11.0	125.4	48.2	12.7	60.0
CD8^+^IFN-γ^+^ recovery (%)	71.1	30.2	70.4	100	87.5	64.0
Viability (%)	51.1	62.1	58.9	52.2	37.2	47.8

*Process performance was evaluated by the recovery (%) and viability (%) of IFN-γ^+^ (CD3^+^/CD4^+^/CD8^+^) T-cells in target (PF) and CMV restimulated before immunomagnetic enrichment (PreS) of clinical-scale CliniMACS CCS^®^ runs with Plus and Prodigy. The viability of cells was assessed by 7-AAD staining and flow cytometry*.

The recovery of IFN-γ^+^ CD4^+^ and CD8^+^ T-cell subsets in the PF as the final product compared to the PreS fractions varied for both instruments. The median recovery was 48.2% (range: 12.7–60.0%) for IFN-γ^+^CD4^+^ T-cells and 87.5% (range: 64.0–100%) for IFN-γ^+^CD8^+^ T-cells on the Prodigy compared to corresponding medians of 69.9% (range: 11.0–125.4%) and of 70.4% (range: 30.2–71.1) on the Plus instrument (Table 3). CD4^+^ to CD8^+^ T-cell ratios reversed during processing from a median of 1.2 (range: 1.02–2.0) in the LA to a median of 0.32 (range: 0.26–6.64) in the PF of the Prodigy processes (Table 1). In the same context, two of three Prodigy products presented higher enrichment ratios for IFN-γ^+^ CD3^+^CD8^+^ T-cells than for the IFN-γ^+^CD3^+^CD4^+^ subset (CD4 to CD8 ratio from 1:5.2 to 1:9.8). Only one process run yielded more IFN-γ^+^ CD3^+^CD4^+^ T-cells (CD4 to CD8 ratio of ≥6.8:1).

### Residual Non-Target Cells in End Products Using Both Plus and Prodigy Procedures

Residual IFN-γ^−^ leukocyte subpopulations were analyzed as known contaminants in each PF and as a quantitative control of recovery also in the NFs derived from both instruments. Contaminating IFN-γ^−^ T-cells ranged from 3.6 to 20.8% (Prodigy) and 18.9 to 80.8% (Plus) among total T-cells in the final products. Accordingly, the median total cell number of IFN-γ^−^CD3^+^ T-cells was 0.04 × 10^6^ (range: 0.01–1.1) with the Prodigy procedure (Table 1) compared to a median of 0.33 × 10^6^ (range: 0.23–0.67) in Plus runs (14). Moreover, all PFs originating from both procedures contained residual CD56^+^CD3^+^ (NK-T) cells and CD56^+^CD3^−^ (NK) cells ≤1.5% gated on viable CD45^+^ cells. However, these contaminants were outnumbered in all PFs of both procedures by residual CD14^+^ monocytes and CD19^+^ B-cells (Prodigy: median [CD14^+^ cells]: 26.2%, range: 11.8–38.0, median [CD19^+^ cells]: 10.8%, range: 4.6–29.0; Plus: median [CD14^+^ cells]: 26.7%, range: 16.3–29.9, median [CD19^+^ cells]: 5.1%, range: 4.9–5.9) for both tested procedures (Figure 4). Absolute cells numbers [×10^6^] of contaminating non-target CD45^+^ leukocyte subsets are represented individually in Figures 4C–F and of IFN-γ^−^ T-cell subsets in Table 1. Finally, CD3 negative cell populations of the PreS, PF, and NF fractions of all Prodigy runs displayed a negligibly low content of less than 0.04% of IFN-γ^+^ cells among B and NKT/NK cells or monocytes.

### Advantages and Disadvantages of the Manufacturing Process Steps for Clinical-Grade CMV-Specific T-Cells by Plus and Prodigy

Figure 1 gives a graphical comparison of the CCS^®^ process flows between the two setups investigated. Ignoring the collection and logistics of the LA and the QCs of starting material and product, the manufacturing sequence can be divided independently of instrumentation into the thrombocyte depletion of the starting material, its incubation under physiological conditions, antigenic restimulation, the consequent IFN-γ secretion and its immediate immunosorbent capture on the IFN-γ^+^ cells’ surface, the paramagnetic immunolabeling of the secreted IFN-γ captured, and the magnetic enrichment of the labeled cells. The process includes frequent washes and solvent exchanges as well as a precise control of temperature profiles and durations – essential during cytokine capture and paramagnetic labeling to prevent a premature saturation of the bispecific cytokine catch matrix (anti-CD45, anti-IFN-γ) and the anti-IFN-γ-labeled dextran-derivatized iron oxide particles by solute IFN-γ that would compromise product purity and process yield. Whereas the Plus procedure’s automation was restricted to the magnetic enrichment step, the Prodigy handled the whole protocol autonomously to a large extent. Given the procedure’s complexity, increased process integration unsurprisingly resulted in substantially reduced hands-on time (approximately 3 man-hours Prodigy vs. approximately 12 man-hours Plus; time for the transfer to and from the clean room, the preparation of media, and the execution of QCs excluded for both setups), rendering irrelevant the increase in system setup due to the Prodigies more complex tubing set (Prodigy, TS 500: 1.5 man-hours vs. Plus, standard: 0.5 man-hours). Our first experiences showed that the Prodigy may allow for single-handed routine operation – unless the four-eyes principle has to be observed, e.g., as a control of the correct installation of the tubing set – whereas up to three qualified operators had to work in parallel on critical and complex offline sub-processes characteristic of the Plus setup. Apart from one optional in-process control (IPC) sampling prior to magnetic separation (PreS), hands-on time for the Prodigy was limited to pre- and post-processing, while scattered over the whole Plus process – leaving out only incubation and antigenic restimulation. Total process duration (Prodigy: approximately 20 h vs. Plus: approximately 26 h, manufacturer’s instructions: ≤36 h) was subject to organizational issues: the regular arrival of the LAs in the afternoons and the restriction to working hours reasonable for one-shift operations resulted in the programing of the Prodigy to 9–11 h of incubation time (manufacturer’s instructions: ≥4 h), allowing for optional IPC sampling already before magnetic enrichment of the target cells at the beginning of the workday. Under the same rationale, the incubation time of the Plus process had to be regularly set to ≥16 h (overnight) due to the fact that even antigenic restimulation required manual initiation. Though not of practical value, process times for both setups were in the same range (approximately 10 h, mandatorily including 4 h of antigenic restimulation) when corrected for this imparity.

## Discussion

Case reports strongly indicate that patients with viral reactivation or *de novo* infection post SCT and SOT refractory to conventional antiviral treatment benefit from the infusion of allogeneic antigen-specific T-cells (2–4). Further investigation requires a rapid and economic supply with adequate doses of this individual cellular therapeutic in clinical grade. During the GMP-compatible establishment of the manufacture of CMV-specific T-cells with the CCS^®^ on the CliniMACS^®^ Plus instrument, we identified the long duration and poor integration of the process as factors possibly compromising these objectives. In parallel to the subsequent GMP-compliant validation of this process (14), we therefore subjected split starting material of three consecutive Plus runs to same procedure on the CliniMACS^®^ Prodigy.

Using the Prodigy reduced the process time to 75% of that necessary for the Plus procedure. A further decrease to 60% would be possible by shortening the initial incubation time toward its specified minimum while simultaneously omitting the only IPC of the Prodigy process (PreS), the latter anyhow dispensable due to the virtual lack of corrective measures at this stage. Lowering the process time from approximately 26 h to approximately 15 h (i.e., overnight) in combination with the simultaneous reduction of hands-on offline time to 25% of that necessary for the Plus process and its restriction to potentially single-handed pre- and post-processing with the Prodigy is also clinically relevant: process automation and integration make up for one working day between apheresis and transfusion, thus either increasing the Prodigies product’s operational shelf life or reducing the time to its application.

From a different angle, automation and integration not consistently backed by IPC and largely inaccessible to manual intervention even in supervised operation necessitate process precision and robustness. In agreement with recent publications (15), our QC results support an adequate degree of standardization.

To compare the two setups, we established a no-wash, single-platform procedure based on 10-color flow cytometry to analyze the cellular composition and viability in process intermediates and by-/products (LA, PreS, PF, and NF) of all three CliniMACS CCS^®^ manufacturing processes.

While the efficiency of restimulation was similar between both instruments by total IFN-γ^+^ T-cells, we found their median recovery (Prodigy: 60.0 vs. Plus: 70.4%) and viability (Prodigy: 47.8 vs. Plus: 58.9%) to be tending lower with the Prodigy but in overall agreement with recent reports (16, 17).

In summary, viabilities as well as viable concentrations and numbers of both target and residual non-target cells in the final product were low but consistent with previous reports (14, 16, 17). The low viability does not result from process-related cell death but is the arithmetic consequence of very low starting frequencies of target cells combined with the unavoidable co-elution of dead cells also unspecifically retained on the tubing set’s column. Therefore, the introduction of an additional washing step to the Prodigies process matrix post-restimulation and labeling, but prior to immunomagnetic enrichment, aiming at the depletion of non-viable target and non-target cells should be considered.

Despite the slightly lower target cell recovery, we observed an increased median purity of the Prodigies products regarding IFN-γ^+^ T-cells among CD3^+^ T-cells (Prodigy: 84.8 vs. Plus: 63.1%) again correlating with other laboratories’ experiences (15–17). However, the observed variability in IFN-γ^+^ T-cell yield with both platforms is indicative for the donor-specific heterogeneity in target T-cell frequency and antigenic responsiveness (Figure 4A), as previously described for variable ranges of purity and recovery of antigen-specific T-cells (15, 16). In our comparative study, both procedures showed a tendency to higher ranges of restimulation frequencies from IFN-γ^+^CD3^+^CD8^+^ T-cells (Plus: 0.3- to 28.6-fold; Prodigy: 5.3- to 122.5-fold) than of IFN-γ^+^CD3^+^CD4^+^ T-lymphocytes (Plus: 0.1- to 4.0-fold; Prodigy: 1.0- to 35.0-fold). Interestingly, two Prodigy runs predominantly yielded IFN-γ^+^CD3^+^CD8^+^ T-cells compared to IFN-γ^+^CD3^+^CD4^+^ cells, whereas the remaining product contained more IFN-γ^+^ helper T-cells than cytotoxic T-cell subsets. Contrary to these results, the percentage of IFN-γ^+^CD3^+^CD8^+^ T-cells was higher than that of IFN-γ^+^CD3^+^CD4^+^ cells in all three Plus validation runs (14). However, similar shifts of IFN-γ^+^ CD4/CD8 ratios in Prodigy target cell products after immunomagnetic separations were reported by Bunos et al. in five Prodigy runs (16), which could be explained by donor-specific differential stabilities/vitalities of restimulated IFN-γ^+^ T-cell subsets after 4 h of antigen incubation. To ensure the efficacy, it is necessary to both stimulate cytotoxic CD8^+^ T-cells and induce a T_helper_-1 (Th1) response persisting *in vivo* as well as the rapid acquisition of appropriate donors and a consistent and well-established record for the manufacturing of virus-specific T-cells with short-term *ex vivo* activation. The latter is aimed at persistent Th1 immunity (18). In summary, we could show that the CCS^®^ produced a mixture of antigen-specific CD4^+^ and CD8^+^ T-cells with both platforms.

Contaminating, potentially alloreactive (IFN-γ^−^) T-cell quantities were less prominent in Prodigy products than in Plus [Prodigy 3.6–20.8 vs. Plus: 19.9–80.0% (14)], which may contribute to improved prevention of the GvHD in recipients. Thus, the Prodigy appears to be at least a platform as adequate for the execution of the CCS^®^ procedure as the less integrated Plus in terms of both purity of antigen-specific T-cells and number of contaminating IFN-γ^−^ T-cells.

Further analyses of residual non-target leukocyte subsets revealed similar low levels of viable CD3^−^CD56^+^ NK cells and CD3^+^CD56^+^ NKT-cells but increased numbers of CD14^+^ monocytes and CD19^+^ B-cells in products of both platforms (Figure 4). One can assume that this carryover of both monocytes/B-cells and subordinate NK/NKT cells is caused by unwanted cross-saturation of catchmatrix molecules bound to those cells by soluble IFN-γ secreted by CMV-responsive T-cells. Given the original frequencies of subsequently contaminating populations in the starting material, one could estimate the order of magnitude of analogous carryover of potentially alloreactive T-cells. Future work should focus on the nature of unspecific capture of contaminants to efficiently reduce residual monocytes and B cells in target cell fractions.

Both platforms allow for the development of various T-cell-based immunotherapies in different indications (19). Unlike applications of unmanipulated donor lymphocyte infusions (DLIs), the application of antigen-specific T-cells reduces the risk of GvHD or other acute side effects while inducing antiviral T-cell reconstitution (8, 20, 21).

In summary, our comparison of Plus and Prodigy showed that both methods can be used for the manufacture of potent CMV-specific T-cells successfully. While the integration and automation of the whole process in one platform promotes process precision and saves hands-on time, the current setup of the Prodigy does not allow for user intervention in case of contingencies. Anyway, unattended operation to the advantage of prolonged product shelf life would make useful interventions virtually impossible in the case of fatal malfunctions affecting, e.g., the process critical temperature management due to reaction times too short – even if the instrument’s potential-free alarm output would be connected to a remote system. Our results support the novel platform’s potential for desired autonomous operation. However, the suitability of this approach has to be justified individually for each process and product irrespective of platform qualification and process validation.

## Conclusion

Although approximately 1 × 10^6^ absolute target cells were collected with both platforms investigated, the product’s cell concentration was consistently below 1 × 10^6^ cells/ml at a fixed low volume of 40 ml. The latter not allowing for extended analysis a multicolor single-platform flow cytometry protocol for comprehensive QC was established. Non-target T-cell quantities were efficiently depleted for prevention of GvHD. Both instruments provided cellular product of comparable characteristics, including antiviral specificity, and may be used alternatively. Independent of the manufacturing platform used and in addition to the flow cytometric QCs presented here, the development of an assay for specific T-cell activity *in vitro* predictive for *in vivo* efficacy (i.e., potency assay) remains a major challenge for this non-cryopreserved cell-poor product regarding assay time, sample size, and clinical relevance.

## Ethics Statement

Informed consent was obtained from all donors as approved by the Ethics Committee of Hannover Medical School.

## Author Contributions

UK, BE-V, and SK designed the study, while CP, RE, and ST were mainly responsible for the performance of the study. More in detail, CP, RE, and MM realized the manufacturing of antigen-specific T-cells. KA, ST, MM, BE-V, UK, and SK carried out the quality control analysis including both cell characterization and the enumeration of the frequency IFN-γ-secreting cells. LG was responsible for the lymphapheresis procedure. CP and SK wrote the manuscript, while BM-K, H-GH, RB, LA, UK, and BE-V contributed to helpful discussions and the careful approval of the final manuscript.

## Conflict of Interest Statement

The authors declare that the research was conducted in the absence of any commercial or financial relationships that could be construed as a potential conflict of interest.
